# Antioxidant and Cytoprotective Potential of the Essential Oil *Pistacia lentiscus* var*. chia* and Its Major Components Myrcene and *α*-Pinene

**DOI:** 10.3390/antiox10010127

**Published:** 2021-01-18

**Authors:** Vasileios Xanthis, Eleni Fitsiou, Georgia-Persephoni Voulgaridou, Athanasios Bogadakis, Katerina Chlichlia, Alex Galanis, Aglaia Pappa

**Affiliations:** Department of Molecular Biology & Genetics, Democritus University of Thrace, 68100 Alexandroupolis, Greece; vasixant4@mbg.duth.gr (V.X.); efitsiou@mbg.duth.gr (E.F.); gvoulgar@mbg.duth.gr (G.-P.V.); abogadak@mbg.duth.gr (A.B.); achlichl@mbg.duth.gr (K.C.)

**Keywords:** essential oil *Pistacia lentiscus* var. *chia*, myrcene, *α*-pinene, antioxidant, wound healing, cytoprotection

## Abstract

The antioxidant, cytoprotective, and wound-healing potential of the essential oil from the resin of *Pistacia lentiscus* var. *chia* (mastic oil) was evaluated, along with that of its major components, myrcene and *α*-pinene. Antioxidant potential was monitored as: (i) direct antioxidant activity as assessed by 2,2-di-phenyl-1-picrylhydrazyl (DPPH), 2,2-azino-bis(3-ethylbenzothiazoline-6-sulfonic acid), and ABTS assays; (ii) DNA damage protection activity; and (iii) cytoprotective activity as assessed via induction of transcription of genes related to the antioxidant response in human keratinocyte cells (HaCaT). The cytoprotective potential of the test substances was further evaluated against ultraviolet radiation B (UVB)- or H_2_O_2_-induced oxidative damage, whereas their regenerative capability was accessed by monitoring the wound closure rate in HaCaT. Μastic oil and major components did not show significant direct antioxidant activity, however they increased the mRNA levels of antioxidant response genes, suggesting indirect antioxidant activity. Treatment of HaCaT with the test substances before and after UVB irradiation resulted in increased cell viability in the cases of pre-treatment with mastic oil or post-treatment with myrcene. Increased cytoprotection was also observed in the case of cell treatment with mastic oil or its major components prior to H_2_O_2_ exposure. Finally, mastic oil and myrcene demonstrated a favorable dose-dependent effect for cell migration and wound closure. Collectively, mastic essential oil may exert its promising cytoprotective properties through indirect antioxidant mechanisms.

## 1. Introduction

The use of plants and their essential oils for therapeutic purposes has been extremely popular since ancient times. Essential oils of various plants appear to have a variety of applications, such as analgesic, antibacterial, antimicrobial, antioxidant, and anticancer applications. They are used by many industries and find applications in agriculture, where they are mainly applied to organic crops as insecticides; in cosmetics to create beauty products; in the sanitary products industry for food preservation and household cleaners; in the food industry to increase the flavor and taste of food; and even in embalmment. Due to the variety of attributed beneficial properties, recent research has turned its interest to studying the bioactivities of aromatic plants and their extracts and further dissecting the involved mechanisms of action [1,2,3,4].

Essential oils are naturally occurring mixtures of volatile, hydrophobic, colorless secondary metabolites. They are synthesized by aromatic plants and they possess characteristic strong aromas. They can be extracted by every plant organ, e.g., from gratia buds, flowers, leaves, stems, twigs, seeds, fruits, root, wood, and bark [5]. Essential oils are mixtures of 20–60 different metabolites in various concentrations, however their quality levels and exact chemical compositions differ substantially in relation to the extraction method, the plant tissue, or even the plant cultivation conditions [1,6,7]. Even though essential oils consist of a variety of compounds, their biological properties are mainly attributed to only a few of their components, usually the ones that are found in the highest concentration (20–70% in comparison with the rest of their components). In general, an essential oil’s composition includes phenols, terpenoids, and flavonoids, exhibiting a wide panel of cytoprotective roles, such as antimicrobial, anti-inflammatory, antifungal, antioxidant, and apoptotic roles [1,7].

*Pistacia lentiscus L*. var *chia* is an endemic cespitose tree grown on Chios Island in the Aegean Sea of Greece. While several species of *Pistacia* are common in the Mediterranean area, *Pistacia lentiscus L*. var *chia* is unique regarding its ability to produce Chios mastic gum (CMG), an aromatic resin used for nearly 2500 years for its health-promoting properties [8]. CMG exhibits increased chemical complexity, being composed of approximately 120 different compounds [9].

Mastic essential oil is a volatile mixture, produced both through traditionally used methods, such as water or steam distillation, or by newly established methodologies, such as supercritical fluid extraction (SFE) [10,11]. The oil is mainly comprised of chemicals categorized as oxygenated monoterpenes, monoterpene hydrocarbons, or sesquiterpenes, which represent approximately 20%, 50%, and 25% of the mastic essential oil, respectively [10,12,13]. Its main constituents are *α*-pinene, myrcene, linalool, and camphene [14]. 

CMG and mastic oil have been associated with a variety of biological functions and have been extensively studied regarding their antimicrobial, anticancer, anti-indigestion, and anti-inflammatory effects [15,16,17]. More particularly, mastic oil has effectively attenuated the development of *Salmonella enteritis*, *Pseudomonas fragi*, *Lactobacillus plantarum*, *Escherichia coli*, *Bacillus subtilis*, and *Staphylococcus aureus* [12,18]. Additionally, its anticancer potential has been demonstrated in a variety of studies. For instance, mastic oil exhibited proapoptotic and antiproliferative properties against the human leukemic cell line K562 in a time- and dose-dependent manner [19]. Accordingly, treatment with mastic essential oil resulted in higher apoptosis, lower vascularization, and downregulation of chemokine expression in an immunocompetent mouse model of Lewis lung carcinoma [20]. The anticancer activity of mastic oil was previously analyzed in colon carcinoma. The oil attenuated the proliferation of a panel of colon cancer cell lines (CT26, Caco-2, HT29), while its oral administration inhibited tumor growth of colon carcinoma in a BALB/c transplantable mouse model. Interestingly, its major components, myrcene and *α*-pinene, did not exhibit the same anticancer activity as the mastic essential oil [17]. 

While several studies describe the health-promoting properties of either CMG or its oil, few data are currently available regarding its antioxidant capacity or its cytoprotective or regenerative effects under oxidative conditions. Oxidative stress plays a leading role in the aging and pathogenesis of degenerative diseases such as atherosclerosis, cardiovascular disease, diabetes, and cancer [21]. Antioxidants can counterbalance the damaging effects of oxidation on tissues and are characterized by their ability to inhibit the production of reactive oxygen species (ROS) or their direct scavenging activity [22]. Through this process, the antioxidants turn themselves into active molecules, but they are much less harmful, preventing significant damage to cell biomolecules [23]. However, antioxidant activity also extends to numerous other cellular physiological processes, such as regulation of signal transduction and regulation of gene expression of detoxifying or antioxidant enzymes [24]. It is also interesting that antioxidants can play a role as pro-oxidants. The same antioxidant molecules that remove active free radicals can cause mild oxidative stress, leading to the induction of cellular signaling to enhance antioxidant defense [25]. Mild stress can turn the level of cellular defense against oxidative stress to a higher steady state, thereby contributing to an adaptive benefit to cell survival. Based on this information, the analysis of the antioxidant activity of bioactive compounds should include the study of all of these fundamental actions [26].

In the present study, we were prompted to investigate the potential direct or indirect antioxidant effects of mastic oil, which was studied for its anticancer properties in a previous report [17], along with its major constituents, myrcene and *α*-pinene. For this reason, we initially evaluated their cell-free antioxidant capacity via DPPH, ABTS, and plasmid DNA protection assays, while we examined their indirect in vitro antioxidant properties by investigating their effect on gene regulation of a panel of genes related to the antioxidant response in human keratinocyte HaCaT cells. Additionally, we assayed the cytoprotective properties of mastic oil, myrcene, and *α*-pinene under H_2_O_2_- and UVB-induced oxidative stress conditions, as well as their healing properties in HaCaT cells.

## 2. Materials and Methods

### 2.1. Essential Oil and Monoterpenes

Mastic essential oil was extracted from the resin of the plant *Pistacia lentiscus* var. *chia* (also known as “mastic gum”) by distillation. Extraction of the oil and the volatile profile analysis was described previously [17]. The oil is a mixture of volatile monoterpenes and a sesquiterpene present at different percentages covering 94.12% of the total chromatographic area. The two most abundant monoterpenes are *α*-pinene and myrcene, accounting for 67.71% and 8.81% of the total chromatographic area, respectively (Table S1) [17]. Along with mastic oil, both these monoterpenes were included in the present study to analyze their antioxidant and cytoprotective potential. *α*-Pinene (90–93%; 0.87 g/mL) was obtained from TREATT (Suffolk, UK), while myrcene (91–93%; 0.81 g/mL) was from TAKASAGO (Tokyo, Japan).

### 2.2. Chemicals and Reagents

Dulbecco’s modified Eagle’s medium (DMEM; high glucose), fetal bovine serum (FBS), trypsin, penicillin-streptomycin, and phosphate-buffered saline (PBS) were purchased from Biosera (Boussens, France). Dimethyl sulfoxide (DMSO), acetic acid, trichloroacetic acid (TCA), Trizma base, sulforhodamine B (SRB), agarose, DPPH, ABTS, and ascorbic acid were purchased from Sigma-Aldrich (BioUltra, Darmstadt, Germany). The pUC19 plasmid DNA was a gift from Joachim Messing (Addgene plasmid # 50005). NucleoZOL was purchased from Macherey-Nagel (Düren, Germany), whereas all primers, dNTPs, random hexamers, and PrimeScript reverse transcriptase were from Invitrogen (ThermoFisher Scientific, Waltham, MA, USA). The KAPA SYBR Fast Master Mix solution was obtained from Kapa Biosystems (Hoffmann-La Roche, Basel, Switzerland).

### 2.3. DPPH and ABTS Assays

The radical scavenging activity was determined using the cell free DPPH and ABTS assays as described previously, with few modifications [27,28]. For DPPH, a range of concentrations (mastic oil: 0.004–45 mg/mL; myrcene: 0.004–40.5 mg/mL; *α*-pinene: 0.004–43.5 mg/mL) were prepared in DMSO. Initial stock solutions of test substances were dissolved in DMSO (1:1 *v/v*). Then, 10 μL of each sample was placed in a 96-well plate and 190 μL of a 300 μM methanolic solution of DPPH was added, while 10 μL of DMSO and 190 μL of DPPH solution were used as the negative control. The plate was then left in darkness for 30 min at room temperature (RT) and the optical density (OD) was subsequently measured at 517 nm using an ELISA plate reader (EnSpire Multimode Plate Reader, Perkin Elmer, Waltham, MA, USA). All experiments were performed in triplicate. The % inhibition of the DPPH radical for each concentration was calculated with the following formula:

% DPPH radical scavenging activity = ((OD_control_ − ODs_ample_)/OD_control_)) × 100.

For the ABTS assay, 7 mM ABTS and 2.45 mM potassium persulfate were mixed in the dark for 16 h to to allow the formation of the ABTS^•+^. Then, 100 μL of the ABTS^•+^ solution was added to 50 μL of the prepared dilutions of mastic oil (0.00445–45 mg/mL), myrcene (0.004–40.5 mg/mL), and *α*-pinene (0.00435–43.5 mg/mL), while 10 μL of DMSO and 190 μL ABTS^•+^ were used as the negative control. The mixtures were incubated for 15 min at RT in the dark and then the absorbance was measured at 734 nm using an Elisa plate reader (EnSpire Multimode Plate Reader, Perkin Elmer, Waltham, MA, USA). The % inhibition of the ABTS^•+^ cation for each concentration was determined by making use of the following formula:

% ABTS^•+^ radical scavenging activity = ((OD_control_ − OD_sample_)/OD_control_)) × 100.

Ascorbic acid was used as a positive control in both DDPH and ABTS assays.

### 2.4. Plasmid DNA Protection Assay

The DNA nicking assay was performed as described earlier with minor modifications [29]. Briefly, 2 μL of plasmid DNA (pUC19) were mixed with 10 μL of Fenton’s reagent (30 mM H_2_O_2_, 50 mM ascorbic acid, and 80 mM FeCl_3_), followed by the addition of different dilutions of mastic oil, myrcene, or *α*-pinene (0.002–2 mg/mL) to a final volume of 20 μL in ddH_2_O. After a 30 min incubation period at 37 °C, bromophenol blue dye (0.25% in 50% glycerol) was added. The reaction mixtures were then loaded on a 0.8% agarose gel and electrophoresis was carried out at 90 V for 1 h followed by ethidium bromide staining. The closed circular, linear, and relaxed forms of pUC19 were visualized and quantified using GelDoc EQ system (Biorad, Segrate, Italy). 

### 2.5. Cell Culture and Treatments

The human immortalized keratinocyte (HaCaT) cell line was purchased by the American Type Culture Collection (ATCC; Manassas, VA, USA). The cell line was maintained in high-glucose DMEM supplemented with 10% fetal bovine serum, 2 mM l-glutamine, and 1% pen/strep (100 U/mL penicillin, 100 μg/mL streptomycin). Cells were cultured in a humidified atmosphere at 37 °C and 5% CO_2_, grown as monolayers, and sub-cultured at approximately 80–90% confluency. 

For the cytotoxicity assays, 10^4^ HaCaT cells were seeded in 96-well plates for 24 h and then treated with a range of concentrations of mastic oil (1.7–222.5 μg/mL), myrcene (1.6–202.5 μg/mL), or *α*-pinene (1.7–217.5 μg/mL) for either 24 h or 48 h, before processing via the SRB assay. Stock solutions of test substances were dissolved in DMSO (1:1 *v/v*) and then serial dilutions were performed in culture medium to achieve the desired range of concentrations. The final concentration of DMSO did not exceed 0.025% (*v/v*). For the evaluation of the cytoprotective effects of the essential oil and its individual components against UVB, HaCaT cells were seeded in 96-well plates 24 h prior to the experiment and subsequently treated with the desired substance at various concentrations (0, 5, 10, 20, 40, 80 μg/mL) for 24 h. Following incubation, cells were UVB-irradiated (55 mJ/cm^2^) and then left to recover for 24 h in fresh medium before proceeding with the SRB assay. In the case of post-treatment with the test substances, HaCaT cells were seeded in 96-well plates for 24 h, their medium was freshly replaced, then they were left to further incubate for another 24 h. Following incubation, cells were UVB-irradiated (55 mJ/cm^2^) and then allowed to recover for 24 h in the presence of the desired substance under a range of concentrations (0, 5, 10, 20, 40, 80 μg/mL) before proceeding with the SRB assay. Finally, for H_2_O_2_ treatment, HaCaT cells were treated 24 h post-plating, either with or without 10 μg/mL of each compound for 24 h. Subsequently, cells were treated with or without 0.2 mM H_2_O_2_ for another 24, before proceeding with the SRB assay. 

### 2.6. SRB Assay

The viability of HaCaT cells was determined using the SRB assay as described earlier, with minor modifications [27,28]. Cells were seeded in 96-well plates and treated with various concentrations of mastic oil, myrcene, or *α*-pinene (1.7–222.5, 1.6–202.5, and 1.7–217.5 μg/mL, respectively) for 24 h or 48 h. Then, cells were fixed by adding 25 μL of 50% *w/v* cold TCA and incubating at 4 °C for 1 h. Subsequently, cells were washed five times with tap water and then stained with 50 μL of 0.4% *w/v* SRB in 1% *v/v* acetic acid for 30 min at RT. Following an additional step of washing with 1% *v/v* acetic acid, the fixed and stained plates were allowed to air dry overnight, followed by solubilization of the bound dye by adding 100 μL of 10 mM Trizma base. Absorbance was measured at 570 nm using an ELISA plate reader (EnSpire Multimode Plate Reader, Perkin Elmer) and the % of cellular viability was estimated using the following formula: ((sample OD_570_ − media blank OD_570_)/(mean control OD_570_ − media blank OD_570_)) × 100. 

### 2.7. Real-Time Polymerase Reaction (RT-qPCR)

Primers for real-time PCR were designed using Primer Express 3.0 software (Applied Biosystems by Life Technologies, Monza, Italy). The gene-specific primers were designed for: β-actin, catalase (CAT), glutathione peroxidase 1 (GPX1), glutathione reductase (GSR), glutathione S-transferase pi 1 (GSTP1), heme oxygenase 1 (HMOX1), nuclear factor erythroid 2-related factor 2 (NRF2), NAD(P)H quinone dehydrogenase 1 (NQO1), and superoxide dismutase (SOD) (Table 1). Total RNA extraction was performed with the NucleoZOL reagent (Macherey-Nagel) according to the manufacturer’s instructions. Then, 1 μg of RNA was used for cDNA synthesis using the SuperScript™ II Reverse Transcriptase (Invitrogen). For real-time PCR experiments, Platinum SYBR Green (Kapa Biosystems) was used according to the manufacturer’s instructions. Real-time PCR reactions were performed in MicroAmp^®^ Fact Optical 48-Well Reaction Plates (ThermoFisher Scientific) using the KAPA SYBR^®^ FAT qPCR Kit (Kapa Biosystems) on an Applied Biosystems Step One instrument (ThermoFisher Scientific) with the following program: 2 min at 50 °C, 2 min at 90 °C, 15 s at 95 °C, and 30 s at 60 °C, with the two last steps repeated for 40 cycles. The melting curve was performed to ensure the absence of primer–dimers or by-products, while for the comparative quantification the ΔΔCt was utilized. Data were normalized to *β*-actin and each reaction was performed in triplicate in three independent experiments.

### 2.8. Wound Healing—Scratch Assay 

A scratch assay was performed as previously described [30]. HaCaT cells were grown in 12-well plates and following confluency were serum-starved and a scratch was introduced with a pipette tip to mimic the formation of a “wound”. After wounding, cells were washed twice with serum-free medium to remove cell debris and treated with different doses of mastic oil, myrcene, or *α*-pinene (0, 5, 10, 20 μg/mL). Photographs of the same fields were taken with a ZEISS Primovert light microscope (Zeiss, Göttingen, Germany) equipped with a digital camera (Axiocam ERc 5 s) at 0 and 12 h. The open wound area was calculated using Image J software (ImageJ; NIH, Bethesda, MD, USA). The relative ratio of wound closure was determined by calculating the ratio of the open surface area at 12 h to that at the initial time of wounding.

### 2.9. Statistical Analysis

At least three independent experiments were performed for each condition tested. The values are expressed as the mean ± standard deviation (SD). Statistical analysis was performed with Graph Pad Prism software (version 8.3.0) (Graph Pad Inc., La Jolla, CA, USA). The concentration of test samples required to cause an inhibition of free radicals by 50% (IC_50_) and the concentration of test samples required to cause a decrease of cell viability by 50% (EC_50_) were calculated from the respective dose–response curves by regression analysis using a four-parameter logistic curve using Sigma Plot Software (v.10) (Systat, San Jose, CA, USA). For all assays, statistical differences between groups were evaluated either by Student’s t-test or ANOVA followed by Dunnett’s or Tukey’s test. A level of *p* < 0.05 was considered statistically significant. 

## 3. Results

### 3.1. Cell-Free Radical Scavenging Activity of Mastic Essential Oil and Its Major Components, Myrcene and α-Pinene 

Τo evaluate the in vitro antioxidant capacity of mastic essential oil and its major components, myrcene and *α*-pinene, the cell-free DPPH and ABTS assays were utilized. Overall, no radical scavenging activity was able to be detected for mastic oil due to turbidity problems that interfered with the assays when the concentrations were increased. Weak radical scavenging activity was observed for myrcene and α-pinene at the higher concentrations, however no accurate IC_50_ values could be determined (Table 2). The IC_50_ for ascorbic acid, which was used as a positive control, was determined to be 26.23 ± 0.16 μΜ by DPPH assay and 30.71 ± 0.24 μM by ABTS assay (Table 2). However, at the highest concentration tested, an inhibitory effect was detected for both myrcene and α-pinene (Table 3). More specifically, *α*-pinene demonstrated weak DPPH and ABTS radical scavenging activity accounting for 47.9 ± 2.78 and 49.28 ± 3.55% inhibition, respectively (Table 3). Myrcene caused only 29.22 ± 6% DPPH inhibition, while no radical inhibition capacity was detected by the ABTS assay (Table 3). 

### 3.2. Antioxidant Capacity of Mastic Essential Oil, Myrcene, and α-Pinene as Monitored by Plasmid DNA Protection Assay

**Figure 1:**
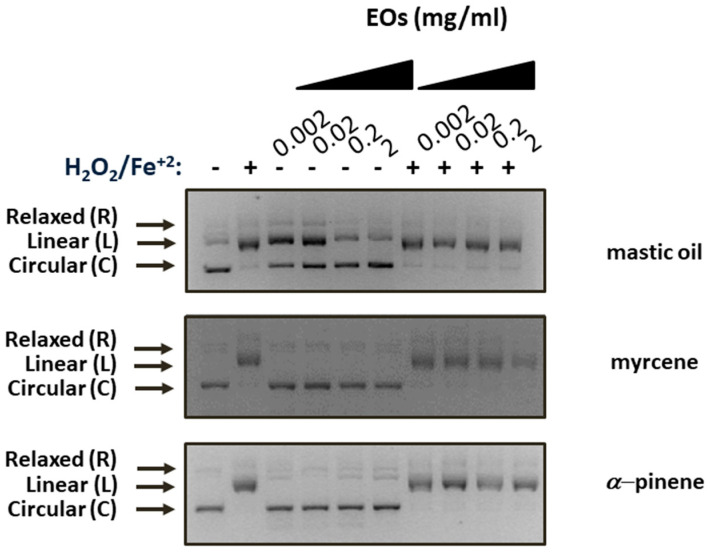
Effects of mastic essential oil and its major components, myrcene and *α*-pinene, on the protection of plasmid DNA pUC19 from H_2_O_2_-induced damage. Lane 1: Plasmid DNA; lane 2: plasmid DNA + Fenton reagent; lanes 3–6: plasmid DNA + mastic oil or myrcene or *α*-pinene; lanes 4–7: plasmid DNA + Fenton reagent + mastic oil or myrcene or *α*-pinene.

The antioxidant potential of mastic essential oil, myrcene, and *α*-pinene was further investigated via the DNA protection assay from damage caused by Fenton’s reaction. This assay aims to evaluate the ability of the test substances to protect plasmid DNA from damage caused by hydroxyl radicals produced via Fenton’s reaction. Hydroxyl radicals are capable of reacting with nucleotides in DNA, causing the formation of cleavages [31]. Plasmid DNA pUC19 was mixed with Fenton’s reagent to induce hydroxyl radicals, and the protection from the genotoxic effects of free hydroxyl radicals was evaluated in the presence of increasing concentrations of mastic oil, myrcene, or *α*-pinene. Control plasmid DNA runs were performed in 3 distinct conformations in gel electrophoresis (relaxed (R), linear (L), and circular (C)), with circular being the predominant form (Figure 1, lane 1). In the presence of the Fenton reagent, the plasmid DNA adopts a supersaturated configuration due to the nicking effect created by the free hydroxyl radicals (L, linear form) (Figure 1, lane 2). Our results indicate that neither mastic oil nor its components, myrcene and *α*-pinene, managed to inhibit DNA oxidation and cleavage under any condition tested (Figure 1, lanes 7–10). The increasing concentrations of mastic oil, myrcene, or *α*-pinene demonstrated no effect on plasmid DNA conformation under non-oxidative conditions either (Figure 1, lanes 3–6). 

### 3.3. Cytotoxicity Profiles of Mastic Essential Oil, Myrcene, and α-Pinene in Human Epidermal Keratinocyte (HaCaT) Cells

**Figure 2:**
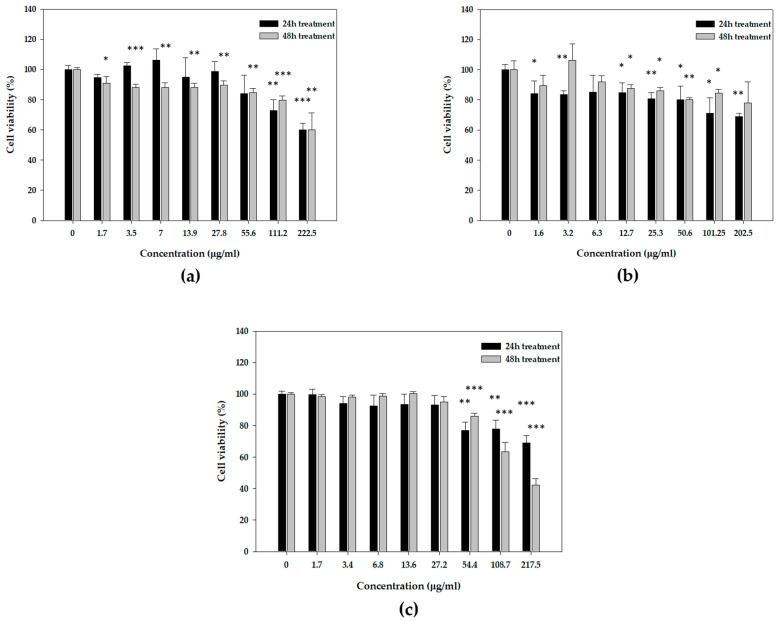
Cytotoxicity effect of (**a**) mastic essential oil and its major components, (**b**) myrcene and (**c**) *α*-pinene, at 24 h or 48 h on HaCaT cells, as determined by the SRB assay. Representative figures of at least three experiments. Results are shown as mean ± SD (*n* = 3). Note: * *p* < 0.05, ** *p* < 0.01, *** *p* < 0.001 vs. control (untreated cells).

In the absence of immediate antioxidant protection, we were prompted to examine the potential enhancement of indirect antioxidant cellular defense caused by these substances. The effects of mastic oil and its major constituents were studied in the human immortalized keratinocyte HaCaT cell line under normal and oxidative stress conditions. HaCaT cells were incubated with increasing concentrations of mastic oil, myrcene, or α-pinene for 24 and 48 h, and cell viability was monitored by the SRB assay. Cell viability curves were plotted to determine the safe range of concentrations to use in subsequent experiments. None of the samples exhibited significant cytotoxicity against HaCaT cells at all the range of concentrations tested. Cytotoxicity was not greater than 50%, with the exception of the 48 h treatment with *α*-pinene at concentrations higher than 100 μg/mL (EC_50_ = 105.5 ± 8.9 μg/mL) (Figure 2). Based on the results from the viability curves, we selected a safe range of concentrations between 5 and 80 μg/mL for all subsequent experiments to ensure that cell viability was greater than 80%.

### 3.4. Effects of Mastic Essential Oil and Its Major Components, Myrcene and α-Pinene, on the Expression Levels of Antioxidant Response Genes

**Figure 3:**
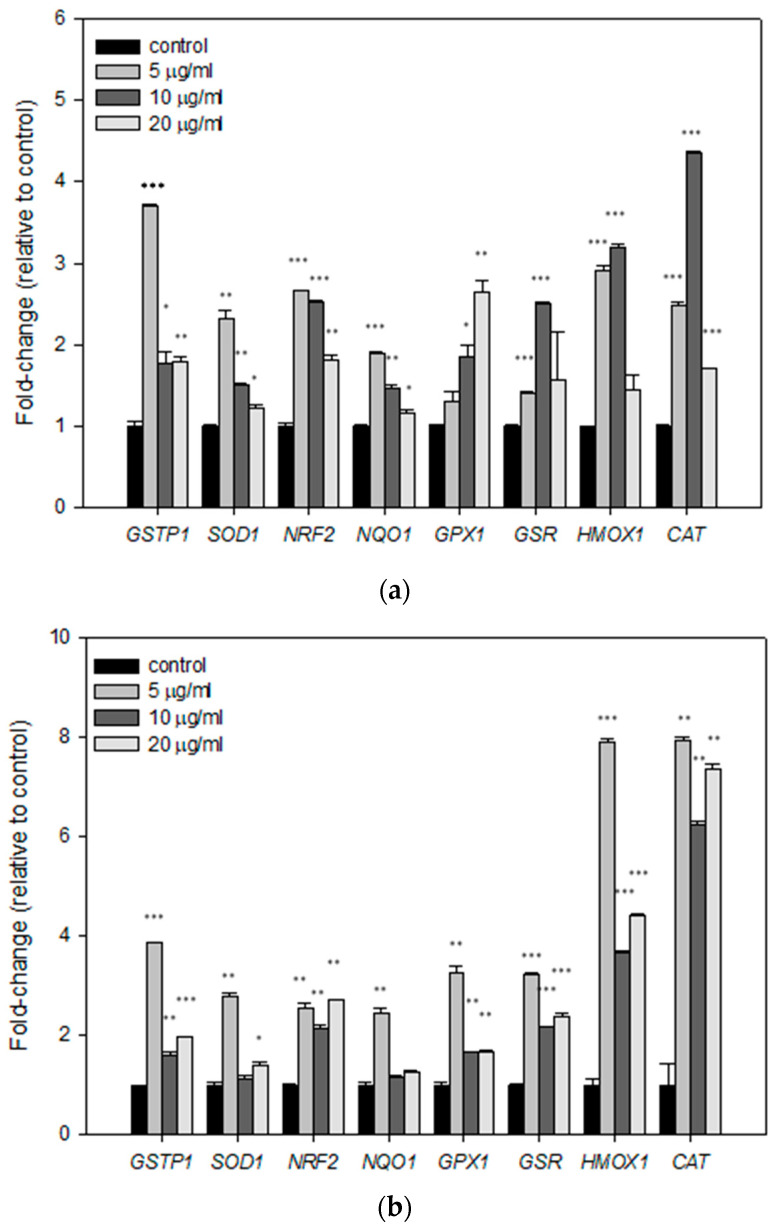

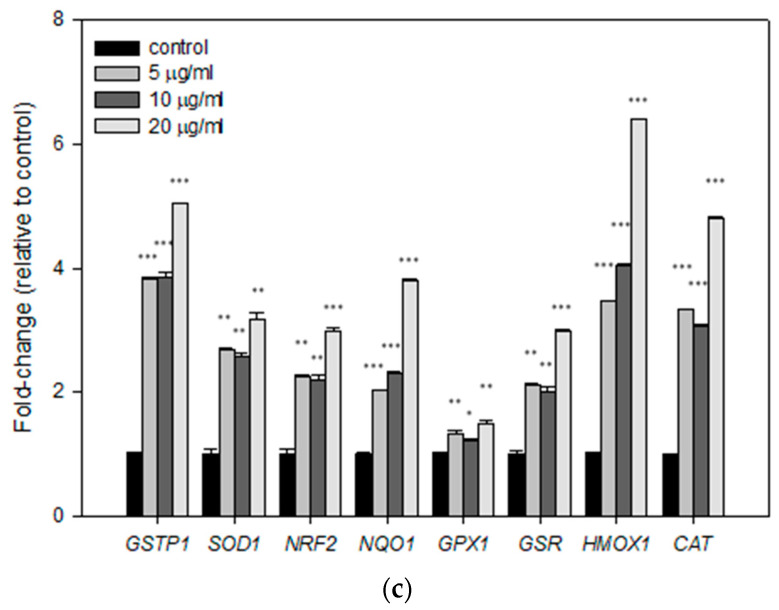
Relative gene expression (mean fold change) of various antioxidant response genes in HaCaT cells treated for 24 h with (**a**) mastic essential oil and its major components, (**b**) myrcene and (**c**) *α*-pinene, as compared to non-treated cells. The comparative quantification method (ΔΔct) was used for the estimation of the fold change of gene expression. *β*-Actin was used as endogenous control to normalize samples. Representative figures of at least three experiments. Results are shown as mean ± SD. Note: * *p* < 0.05, ** *p* < 0.01, *** *p* < 0.001 vs. control (untreated cells).

Next, we examined whether treatment with the mastic oil, myrcene, or *α*-pinene could affect gene regulation of a panel of genes related with the cellular antioxidant response mechanisms. For this purpose, 10^6^ HaCaT cells were seeded in 100 mm plates and treated with 5, 10, or 20 μg/mL mastic oil, myrcene, or *α*-pinene for 24 h. Cells were then harvested and their total RNA was isolated in order to synthesise cDNA and perform comparative real-time PCR for the genes of interest. Our results showed that treatment with all test substances resulted in significant upregulation of *NRF2* transcription factor, as well as its target genes *GSTP1*, *SOD1*, *NQO1*, *GPX1*, *GSR*, *HMOX1*, and *CAT*. Specifically, in the case of treatment with mastic oil, maximum upregulation was achieved with the lowest concentration used (5 μg/mL) for expression of *GSTP1*, *SOD1*, *NRF2*, and *NQO1*, while a dose-dependent increase was observed for the expression of *GPX1* (Figure 3a). The lowest concentration of myrcene also had the same effect, causing the maximum increase in the mRNA levels of all genes studied (Figure 3b). In the case of treatment with *α*-pinene, a dose-dependent effect was apparent for the expression of most of the genes studied (Figure 3c). 

### 3.5. Cytoprotective and Regenerative Effects of Mastic Oil and Its Major Components on HaCaT Cells after UVB Irradiation

**Figure 4:**
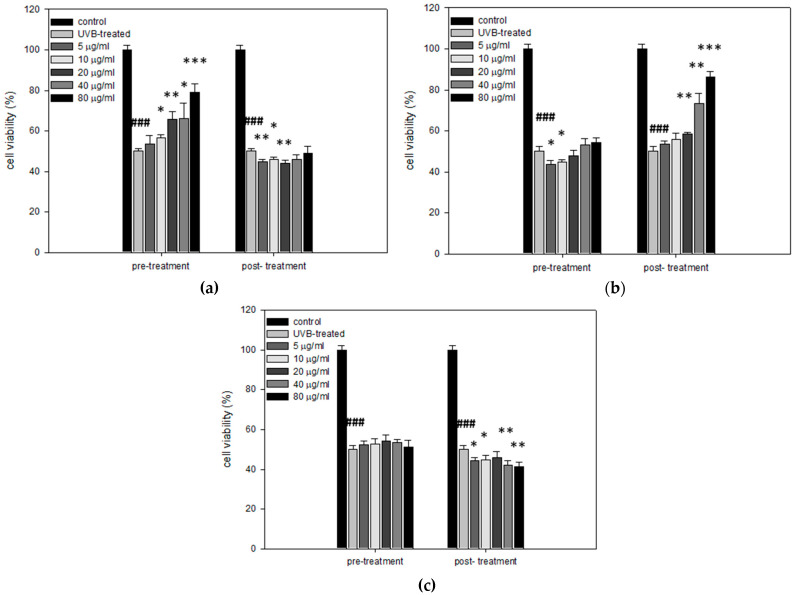
Viability of HaCaT cells pre- or post-treated with (**a**) mastic essential oil, (**b**) myrcene, or (**c**) *α*-pinene under conditions of UVB exposure. Representative figures of at least three experiments. Results are shown as the mean ± SD. Note: * *p* < 0.05, ** *p* < 0.01, *** *p* < 0.001 vs. UVB-treated cells, ### *p* < 0.001 vs. control (untreated cells).

Next, the potential cytoprotective and regenerative effects of mastic oil, myrcene, and *α*-pinene against UVB radiation were assessed. HaCaT cells were seeded in 96-well plates and treatments with mastic oil, myrcene, or *α*-pinene were performed in two different experimental setups, following a pre-treatment or a post-treatment protocol. In the case of the pre-treatment protocol, HaCaT cells were treated with 0, 5, 10, 20, 40, or 80 μg/mL mastic oil, myrcene, or *α*-pinene for 24 h. Then, cells were irradiated with 55 mJ/cm^2^ UVB to induce a drop in cell viability at approximately 50% [19] and allowed to recover for 24 h. Subsequently, their viability was estimated by SRB assay. In the case of the post-treatment protocol, cells were irradiated with 55 mJ/cm^2^ UVB radiation and then treated for 24 h with 0, 5, 10, 20, 40, or 80 μg/mL mastic oil, myrcene, or *α*-pinene. After the 24 h post-treatment, their viability was estimated via SRB assay. Our results indicate that pre-treatment with mastic oil significantly increased the cell viability of HaCaT cells in a dose-dependent manner (Figure 4a). However, post-treatment with mastic oil resulted in no significant protection effect (Figure 4a). The opposite pattern was observed for myrcene. Specifically, pre-treatment of HaCaT cells with myrcene prior to UVB irradiation had no effect on their viability, while post-treated cells retained their viability in a statistically significant and dose-dependent manner (Figure 4b). Finally, *α*-pinene did not exhibit any cytoprotective effect, neither when applied before nor after UVB irradiation in HaCaT cells (Figure 4c).

### 3.6. Cytoprotective Effects of Mastic Essential Oil, Myrcene, and α-Pinene against H_2_O_2_ Cytotoxicity in HaCaT Cells

**Figure 5:**
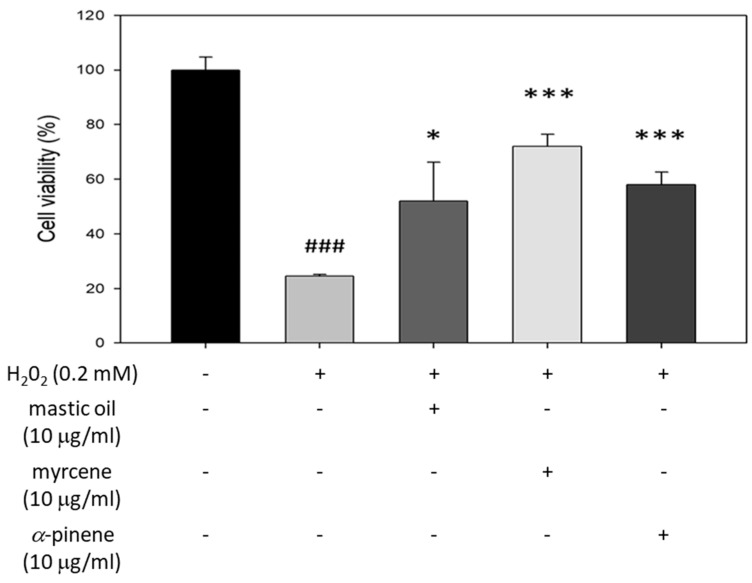
Cytoprotective effects of mastic essential oil and its major components, myrcene and *α*-pinene, against H_2_O_2_-induced oxidative damage in HaCaT cells. Representative figures of at least three experiments. Results are shown as the mean ± SD. Note: * *p* < 0.05, *** *p* < 0.001 vs. H_2_O_2_-treated cells, ### *p* < 0.01 vs. control (untreated cells).

Mastic essential oil and its major components myrcene and *α*-pinene were also studied regarding their potential to protect HaCaT cells against a known oxidative agent (H_2_O_2_). HaCaT cells were seeded in 96-well plates and treated with 10 μg/mL of mastic oil, myrcene, or *α*-pinene for 24 h. Then, cells were washed with PBS and incubated with culture media with or without 0.2 mM H_2_O_2_ for 24 h. Following H_2_O_2_ treatment, cell viability was determined via SRB assay. Treatment with H_2_O_2_ resulted in significant loss of cell viability (Figure 5). Pre-incubation for 24 h with either mastic oil or its major components significantly increased cell viability when compared to HaCaT cells treated only with H_2_O_2_. The highest protective effect was demonstrated by myrcene, followed by *α*-pinene and mastic oil (Figure 5). 

### 3.7. Effects of Mastic Essential Oil and Its Major Components, Myrcene or α-Pinene, on Migration and Regeneration of HaCaT Cells

**Figure 6:**
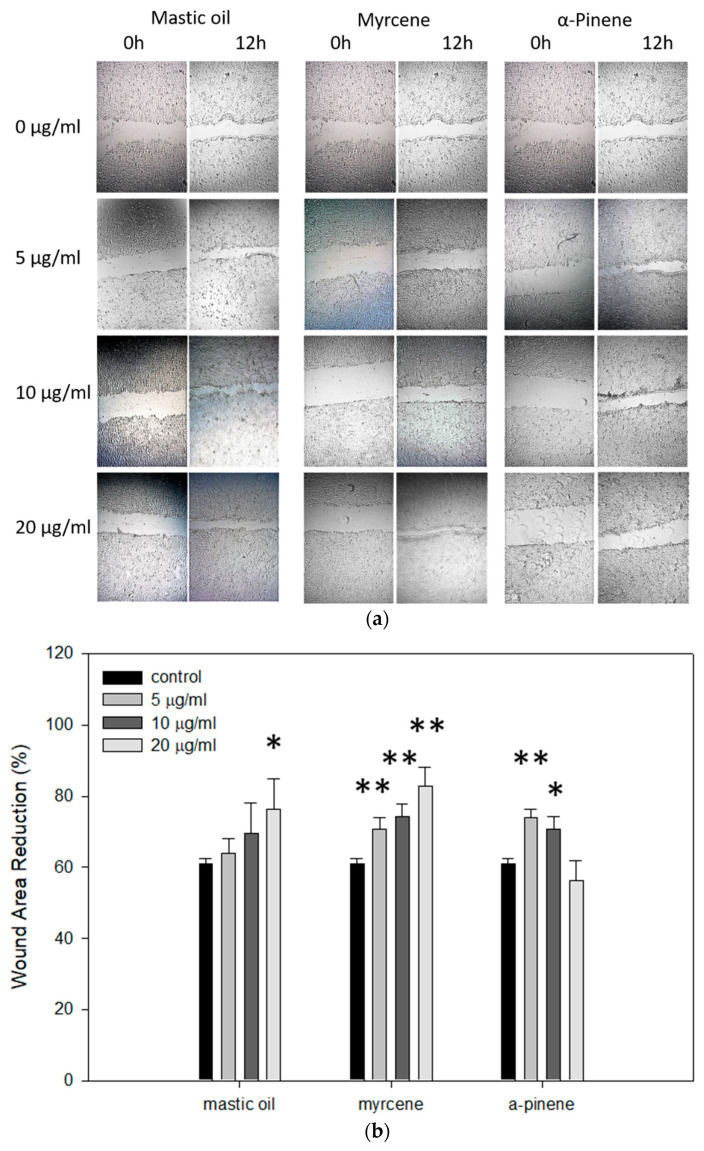
Effects of mastic essential oil and its major components, myrcene and *α*-pinene, on migration and regeneration of HaCaT cells. (**a**) Migration of cells was monitored by successive images under an optical microscope taken at the indicated time points. (**b**) Quantification of the percentage of wound closure by ImageJ software analysis. Data are presented as the mean ± SD of three independent experiments. Note: * *p* < 0.05, ** *p* < 0.01 vs. control (untreated cells).

Finally, we investigated whether mastic oil, myrcene, or *α*-pinene has the potential to induce healing using the scratch assay. Our data demonstrate that in the presence of mastic oil or myrcene, the wounds were healed more rapidly compared to control (Figure 6a), displaying a dose-dependent effect (Figure 6b). Treatments with *α*-pinene also resulted in faster recovery, however not in a dose-dependent manner (Figure 6a,b). 

## 4. Discussion

The resin of *Pistacia lentiscus* var. *chia* (mastic gum) has been known since antiquity, with many uses in cosmetics, food flavoring, and medicine. It is a unique Greek product produced exclusively on the island of Chios. The mastic essential oil is quite popular and has regained interest as a potent phytotherapeutic agent due to numerous studies documenting a variety of health-promoting properties [9]. Mastic essential oil was extracted from the resin produced by the mastic tree as a trunk exudate by direct gum distillation. The oil is mainly composed of volatile terpenes, with gas chromatography–mass spectroscopy (GC-MS) analysis identifying several components, with *α*-pinene and myrcene being the most abundant ones, accounting for 67.71% and 18.81%, respectively (Table S1). 

In the present study, we investigated the antioxidant and cytoprotective potential of the essential oil derived from the resin of *Pistacia lentiscus* var. *chia* [17] and its two major constituents, myrcene and *α*-pinene. Regarding their direct antioxidant activity, our results indicated that neither mastic oil nor its constituents exhibit considerable radical scavenging activity in the DPPH and ABTS assays, while they could not protect plasmid DNA from Fenton’s reaction-induced oxidative damage. Similar observations were reported in a study where the mutagenic and antimutagenic potential of Chios mastic oil was accessed, showing that mastic oil could not afford any protection against DNA damage induced by mitomycin C in human lymphocytes using the cytokinesis block micronucleus assay [32]. On the other hand, myrcene was found to be ineffective in reducing DNA damage in HepG2 cells following exposure to (tert-butyl hydroperoxide) t-BOOH [33]. The data in the literature are controversial concerning the direct antioxidant activity of the two terpenes. Although *α*-pinene indeed shows increased antioxidant activity compared to myrcene, their potency varies from undetectable to significant, depending on the study [34,35,36]. As far as mastic preparations are concerned, the data are currently scarce regarding the direct antioxidant properties. For instance, Triantafyllou et al. showed that CMG (containing 40–55% triterpenic acids and 20–25% polymer fraction (poly-β-myrcene)) did not exhibit direct scavenging potential against superoxide in a xanthine oxidase system, but it was able to inhibit tumor necrosis factor α (TNF-α)-induced production of H_2_O_2_ and superoxide in aortic rat smooth muscle cells and to attenuate protein kinase C (PKC) activity, suggesting indirect antioxidant mechanisms [37]. In another study, it was demonstrated that CMG could efficiently inhibit the copper-induced oxidation of low-density lipoprotein (LDL) in vitro [38], but rather than solely counteracting with the oxidized LDL (oxLDL), it was pointed out that the resin enhances cell defenses via pivotal physiological pathways, e.g., by downregulating CD36 and maintaining glutathione levels in peripheral blood mononuclear cells (PBMCs). In this way, the total polar extract of the resin used in the study exerted its antiapoptotic and antioxidant roles and protected the PBMCs from oxLDL-induced oxidative stress and necrosis [39]. 

Besides *Pistacia lentiscus* from Greece (var. *chia*), there are other reports on *Pistacia lentiscus* species grown in other parts of the world. In Tunisian *Pistacia lentiscus*, the most prevalent constituents identified at the whole-plant level were *α*-pinene, *β*-myrcene, *α*-limonene, and germacrene-D. Differences were also observed in the essential oil and phenol contents extracted from the plant, which were reported to be season- and gender-related, as well as plant-part-related within the same gender. Remarkable antioxidant activity monitored by DPPH and ferric-reducing antioxidant power (FRAP) assays was demonstrated for the acetonic extracts from both plant genders in the Tunisian species [40]. In another study, Bouyahya et al. reported that the volatile compounds of *Pistacia lentiscus* L. (Morocco) essential oil extracted from the fruits of the plant had better free radical scavenging and ferric-reducing properties compared to the essential oil extracted from the plant leaves [41]. The GC-MS analysis identified 22 compounds, with the main components being myrcene (33.5%) and *α*-pinene (19.2%) in the essential oil extracted from the plant leaves, while limonene (18.3%) and α-pinene (20.5%) were the main components in the essential oil extracted from the plant fruits. Although there are differences in the chemical composition between the mastic preparations used across the studies, a recent study characterized and compared the volatile oils of the mastic gum and mastic oil and revealed that α-pinene and myrcene are the two major components found in the two mastic preparations [42]. From the above information, it becomes apparent that the antioxidant activity reported for *Pistacia lentiscus* is variable to many factors, such as the chemical composition of the extracts prepared, the kind of the plant parts used, the plant gender, as well as the species variance, collecting season, and geographic origin. 

Interestingly, our results demonstrated that even though mastic oil and its two main constituents demonstrated only negligible cell-free radical scavenging activity, they exhibited significant indirect antioxidant capacity by upregulating a set of genes associated with antioxidant cellular response in human keratinocytes. Incubation of HaCaT cells with mastic oil, as well as myrcene or α-pinene, resulted in significant upregulation of the *NRF2* transcription factor and its target genes *GSTP1*, *NQO1, GPX1*, *GSR* (coding for detoxifying enzymes), *SOD*, *HMOX1*, and *CAT* (coding for antioxidant enzymes). In most cases, this upregulation occurred in a dose-dependent and statistically significant manner, suggesting that mastic oil may exert its antioxidant properties through indirect mechanisms. NRF2 is a central regulator of the adaptive cellular response to oxidative stress. Under normal conditions, NRF2 is constitutively expressed at low levels, being repressed mainly by the Kelch-like ECH-associated protein 1 (KEAP1) through ubiquitination and proteasomal degradation. When NRF2 is activated in response to oxidative stress, it gets released from KEAP1, thereby avoiding degradation, and is translocated to the nucleus, where it binds to the antioxidant response element (ARE) in the promoter regions of various detoxifying and antioxidant enzymes, amongst others [43,44,45]. These groups of enzymes are normally important for maintaining the redox homeostasis of the cell and reducing the excess amount of ROS, thus preventing cellular oxidative damage [35,36]. Many natural products and small molecules have been characterized as NRF2 activators and exert their cytoprotective properties (neuroprotective, hepatoprotective, skin protective, and chemoprotective properties, etc.) through regulating the NRF2-Keap1-ARE signaling pathway [46,47,48,49].

The cytoprotective potential of mastic oil, myrcene, and α-pinene was studied against the oxidative agents UVB and H_2_O_2_, which induce cellular oxidative stress via different mechanisms. UVB is a potent oxidative agent and exposure to UVB radiation leads to the generation of ROS from the excitation of O_2_ to form singlet oxygen, along with the transfer of 1, 2, or 3 electrons to oxygen to form superoxide, hydrogen peroxide, and hydroxy radical, respectively [50], resulting in oxidative damage to membrane lipids, nucleic acids, and proteins, causing cellular destruction [51]. The oxidative stress achieved by H_2_O_2_ treatment utilizes H_2_O_2_ as a precursor to hydroxyl radical, which is less reactive but more readily diffusible, and thus more likely to be involved in the formation of oxidized bases [52]. Our results showed that pre-treatment with mastic oil or post-treatment with myrcene prior to or following UVB irradiation, respectively, resulted in increased viability of HaCaT cells. On the contrary, α-pinene could not protect HaCaT cells from the cytotoxic effects of UVB radiation. Although the differential cyto-protection observed is quite interesting, it cannot be explained in detail yet, but may provide the basis for further testing of mastic oil and myrcene in applications such as skin protective agents against UV radiation. The specificity of the chemical composition of the oil (the potential synergistic effect of the mixture), differences in the UV-absorbing spectra (acting as UV blockers), or possible differential effects on the efficiency of DNA repair mechanisms caused by the compounds may explain the differences observed, and further research is required towards this direction. On the other hand, all tested compounds managed to efficiently protect HaCaT cells from the cytotoxic effects of H_2_O_2_, which may be explained by the different mechanisms of action that the two oxidants exert. To the extent of our knowledge, this is the first time that the cytoprotective properties of mastic oil under oxidative stress conditions are being reported. We hypothesize that mastic oil and its constituents could exert their cytoprotective effect through inducing NRF2 and other genes associated with antioxidant response, thus enhancing the cellular defense under oxidative conditions. Similarly, the monoterpenes α-pinene and 1,8-cineol were reported to exert neuroprotective potential against H_2_O_2_-induced oxidative stress in PC12 cells by eliciting an antioxidant response through induction of the nuclear NRF2 factor and upregulation of the antioxidant enzymes CAT, SOD, GPX1, GSR, and HMOX1 [53]. 

In a different study, the gene expression profile of zebrafish fed with a mastic-oil-supplemented diet using microarray analysis revealed that mastic oil caused differential expression of interferon-response-related genes, upregulation of the immune responsive gene 1 (*irg1*), and increase of *mucin 5.2*, which is involved in host defense against pathogens [54]. These results further support the notion that the effects of mastic oil on gene transcriptional activation may regulate its beneficial actions. In the present study, however, whether mastic oil exerts its antioxidant effects either by acting as a pro-oxidant or by affecting the transduction of other signaling pathways that are involved in the alleviation of oxidative stress was not investigated and requires further research. The biochemical and molecular mechanisms that are related with the antioxidative action of *Pistacia lentiscus*, as discussed here, appear to be quite diverse. As mentioned above, it is of the utmost importance to correlate the antioxidant activity reported with the chemical analyses of the terpene and phenol contents of the plant parts or the tree resin, taking into consideration the species variation, gender, and developmental stage of *Pistacia lentiscus*.

Finally, the potential wound healing properties of mastic oil and its major constituents were studied via the in vitro scratch assay. Our data indicated that Chios mastic oil, myrcene, and α-pinene exhibit promising migratory and regenerative properties in HaCaT cells. To the extent of our knowledge, this is the first time that the wound healing potential of mastic oil, myrcene, or α-pinene is being reported. There are scarce reports in the literature associating mastic gum with increased reinforcement of surgical adhesive strips [55,56,57]. Recently, the European Medicines Agency (EMA) recognized mastic gum as a traditional herbal medicinal product with the therapeutic indication of skin inflammation and healing of minor wounds [9]. However, the exact mechanism(s) that underlie the wound healing properties of mastic resin or essential oil remain to be elucidated. It is quite interesting that various bioactive compounds that act as NRF2 inducers also accelerate the wound healing process [58]. Whether there is an association between the upregulation of the NRF2-ARE pathway by mastic essential oil and its wound healing properties remains to be elucidated. 

The bioavailability of mastic essential oil or its major components has not been thoroughly investigated. However, there are several reports that suggest that the terpenes, α-pinene, and myrcene present in the mastic oil are absorbed by humans and demonstrate their bioavailability pattern following oral administration [59,60]. More specifically, blood samples from human volunteers were collected at different time points before and after oral consumption of mastic oil. Following monitoring by GC-MS, it was determined that α-pinene and myrcene significantly increased following 0.5 h of essential oil administration and remained significantly increased at 1, 2, 4, 6, and 24 h. An increase in serum lipid resistance to oxidation was also observed, which was significant at 1 to 4 h and peaked at 2 h after consumption [60]. In addition, both monoterpenes, myrcene and α-pinene, are absorbed by the skin [61], and in general terpenes are well recognized and relatively safe skin penetration enhancers for drug permeation across the human skin and have been receiving considerable attention for this property in pharmaceutical applications [62]. 

Ιn comparison with other species, similar antioxidant biological activity has been reported for the essential oils of *Sideritis raeseri* subps. *raeseri, Citrus sinensis*, and *Citrus latifolia*. *Sideritis raeseri* essential oil possessed weak radical scavenging activity but demonstrated substantial cytoprotective activity against H_2_O_2_-induced oxidative stress and DNA damage in HaCaT cells. The main components of the oil were geranyl-*p*-cymene (25.08%), geranyl-*γ*-terpinene (15.17%), and geranyl-linalool (14.04%), but α-pinene (2.13%) and caryophyllene (2.88%) are common with mastic essential oil [63]. The essential oils of *Citrus sinensis* and *Citrus latifolia* were found to efficiently decrease apoptosis in HaCaT cells following exposure to H_2_O_2_ and to lower intracellular superoxide ion. R-(+)-limonene, *β*-thujene, α-myrcene, and *γ*-terpinene were identified as their major components [64]. However, α-pinene and myrcene are also present, but are found in minimal abundance in the *Citrus* essential oils. In another study by Haghdoost et al., the essential oil of *Pistacia atlantica* resin extract demonstrated a concentration-dependent effect on the healing of burn wounds through increasing basic fibroblast growth factor (bFGF) and platelet-derived growth factor (PDGF) and enhancing angiogenesis. Quite interestingly, *α*-pinene (46.57%) was identified as the main constituent of the essential oil [65]. Detailed studies on the potencies and the capacities of the single components of essential oils, as well as their synergy effects, are required to reach more accurate conclusions on the relation between biological activity and chemical composition. 

## 5. Conclusions

**Figure 7:**
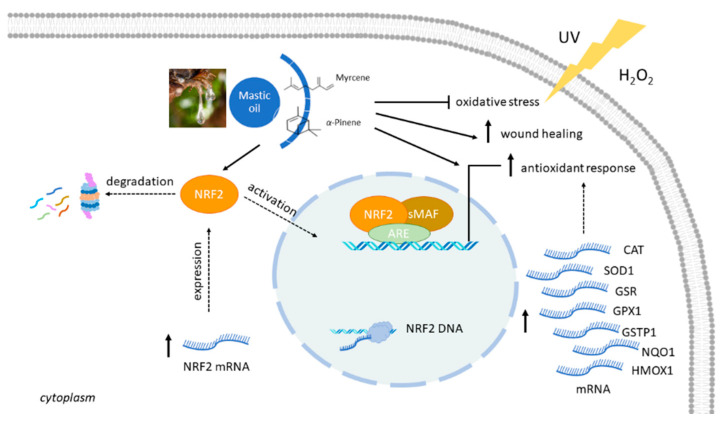
Proposed mechanisms of the antioxidative actions of *Pistacia lentiscus* var. *chia.* Mastic essential oil confers a cytoprotective effect against H_2_O_2_- and UV-induced oxidative stress in skin epithelial cells by inducing *NRF2* mRNA expression and subsequently upregulating the Nrf2–ARE pathway, which plays a fundamental role in maintaining cellular redox homeostasis. Under normal conditions, NRF2 is constitutively expressed at low levels, being repressed mainly by KEAP1 through ubiquitination and proteasomal degradation. When NRF2 is activated in response to oxidative stress it gets released from KEAP1, thereby avoiding degradation, and is translocated to the nucleus, where it binds to ARE along with the small musculoaponeurotic fibrosarcoma protein (sMaf) in the promoter regions of various antioxidant response proteins and phase II detoxifying enzymes. Activated ARE-driven downstream antioxidant defense mechanisms will lead to the restoration of normal physiological conditions and to the alleviation of oxidative stress. We propose that mastic essential oil activates the Nrf2–ARE pathway by inducing *NRF2* expression, which potentially influences the pathway at different stages (e.g., expression, degradation, and activation), leading ultimately to the upregulation of the pathway and the transcriptional activation of antioxidant response proteins. The exact details of the mechanisms remain unknown and require further investigation (dashed lines). Mastic essential oil also exerts enhanced cell regeneration and wound healing actions.

Overall, the results of the present study indicated that neither mastic oil, nor its major constituents, *α*-pinene and myrcene, exhibited direct radical scavenging activity, as demonstrated by DPPH, ABTS, and plasmid DNA protection assays. However, both the oil and its major components induced alterations in the gene expression profile of the oxidative response-related genes *NRF2*, *GSTP1*, *SOD1*, *NQO1*, *GPX1*, *HMOX1*, and *CAT* in HaCaT cells, suggesting indirect mechanisms of their antioxidative activities. Under conditions of oxidative stress, all test substances demonstrated increased cytoprotective properties against H_2_O_2_, while treatment with mastic oil or myrcene prior to or post UVB exposure, respectively, led to enhanced cell viability in HaCaT cells. Moreover, all tested compounds demonstrated enhanced regenerative wound healing capacities. The proposed mechanisms of the antioxidative actions of *Pistacia lentiscus* var. *chia* are summarized in Figure 7. Our results provide strong support for the promising candidacy of mastic essential oil for future pharmaceutical applications. However, further studies are required, which are currently underway, to elucidate in more detail the mode(s) and mechanism(s) of action of mastic oil, and also to gain insight into the structure–function relationship and potential synergy effects behind its properties and potencies.

## Figures and Tables

**Table 1:** List of primer sequences used for Real time PCR.

Gene	Forward Primer (5′→3′)	Reverse Primer (5′→3′)	Accession Number	
*β-actin*	GCGCGGCTACAGCTTCA	CTTAATGTCACGCACGATTTCC		NM_001101.5
*CAT*	ACATCTGAAGGATCCGGACA	ATGCAGAGACTCAGGACGTA		NM_001752.4
*GPX1*	GGCAAGGAGAACGCCAAGA	AGCATGAAGTTGGGCTCGAA		NM_001329455.2
*GSR*	GAGGTGCTGAAGTTCTCCCA	TGACTTCCAAGCCCGACAAA		NM_000637.5
*GSTP1*	TGGTGGACATGGTGAATGAC	AGATGTATTTGCAGCGGAGG		NM_000852.4
*HMXO1*	CAGTCAGGCAGAGGGTGATA	CTCCTCAAAGAGCTGGATGTT		NM_002133.3
*NRF2*	CAGCTTTTGGCGCAGACATT	AAGTGACTGAAACGTAGCCGA		NM_006164.5
*NQO1*	CCAGAAAGGACATCACAGGTAA	AGACTCGGCAGGATACTGAA		NM_001025434.2
*SOD1*	GAGACCTGGGCAATGTGACT	GTTTACTGCGCAATCCCAAT		NM_000454.5

**Table 2:** Antioxidant activity of mastic oil and its major components, myrcene and α-pinene, using the DPPH and ABTS assays. Data are presented as the mean ± SD of IC_50_ values determined in at least three independent assays. Triplicate measurements were conducted in each assay. Ascorbic acid was used as a positive control.

IC_50_ (μM)		
	DPPH	ABTS
Mastic oil	n.d.	n.d.
Myrcene	n.d.	n.d.
*α*-Pinene	n.d.	n.d.
Ascorbic acid	26.13 ± 0.16	30.71 ± 0.24

Note: n.d. = not determined.

**Table 3:** Antioxidant activity of myrcene and *α*-pinene at the highest concentration tested using the DPPH and ABTS assays. Data are presented as the mean ± SD of at least three independent experiments.

Inhibition (%)		
	DPPH	ABTS
Myrcene (40.5 mg/mL)	29.22 ± 6	0
*α*-Pinene (43.5 mg/mL)	47.9 ± 2.78	49.28 ± 3.55

## Data Availability

The data presented in this study are available within the article and its supplementary material. Other data that support the findings of this study are available upon request from the corresponding authors.

## Supplementary Materials

The following are available online at https://www.mdpi.com/2076-3921/10/1/127/s1, Table S1: The volatile compounds identified in *Pistacia lentiscus* var. *chia* essential oil by GC/MS analysis and their relative percent (%) chromatographic area.
