# Supplementary material for: Antioxidant and Cytoprotective Potential of the Essential Oil Pistacia lentiscus var. chia and Its Major Components Myrcene and α-Pinene
Source: Antioxidants (Basel). 2021 Jan 18;10(1):127. doi: 10.3390/antiox10010127 (PMC7830477; doi:10.3390/antiox10010127)
Supplement: Supplementary file 1 [file antioxidants-10-00127-s001.pdf]

Antioxidant and Cytoprotective Potential of the Essential Oil *Pistacia lentiscus* var. *chia* and Its Major Components Myrcene and *α*-Pinene

Vasileios Xanthis, Eleni Fitsiou, Georgia-Persephoni Voulgaridou, Athanasios Bogadakis, Katerina Chlichlia, Alex Galanis * and Aglaia Pappa *

Supplementary Material

Table S1. The volatile compounds identified in *Pistacia lentiscus* var. *chia* essential oil by GC/MS analysis and their relative percent (%) chromatographic area. Data adapted from [17].

KRI*	Compounds	% Area
920	*α*-pinene	67.71
976	myrcene	18.81
958	*β*-pinene	3.05
1010	limonene	0.89
1086	linalol	0.73
934	camphene	0.70
1120	*cis*-verbenol	0.69
1405	caryophyllene	0.5
1113	pinocarveol	0.32
1168	verbenone	0.32
1094	*α*-campholenic aldehyde	0.26
937	verbenene	0.07
1117	*trans*-verbenol	0.07

* KRI: Kovats Retention Indices.
